# Risk and Protective Factors of Self-harm and Suicidality in Adolescents: An Umbrella Review with Meta-Analysis

**DOI:** 10.1007/s10964-024-01969-w

**Published:** 2024-04-02

**Authors:** Rebecca Richardson, Tanya Connell, Mandie Foster, Julie Blamires, Smita Keshoor, Chris Moir, Irene Suilan Zeng

**Affiliations:** 1 Faculty of Health and Environmental Science, Research Office, Department of Biostatistics and Epidemiology, Auckland University of Technology, Auckland, New Zealand; 2 Faculty of Culture and Society, School of Social Sciences and Public Policy, Auckland University of Technology, Auckland, New Zealand; 3 Faculty of Health and Environmental Science, School of Nursing, Auckland University of Technology, Auckland, New Zealand; 4 School of Midwifery and Nursing, Edith Cowan University, Perth, WA Australia; 5 Faculty of Health and Environmental Science, School of Oral Health, Auckland University of Technology, Auckland, New Zealand; 6 Centre for Postgraduate Nursing Studies, University of Otago, Christchurch, New Zealand

**Keywords:** Risk and Protective factors of suicidality, Risk and Protective factors of non-suicidal self-harm, Vulnerable adolescent, Bully victimization, Sleep disturbance, Umbrella review, Meta-analysis

## Abstract

Suicide remains the second most common cause of death in young people aged 10–24 years and is a growing concern globally. The literature reports a vast number of factors that can predispose an adolescent to suicidality at an individual, relational, community, or societal level. There is limited high-level research identifying and understanding these risk and protective factors of adolescent suicidality. The present study used an umbrella review and meta-analysis to synthesize evidence from the review literature in the past 20 years on risk and protective factors of self-harm and suicidality (behavior and ideation) in adolescents. The umbrella review included 33 quantitative reviews with 1149 individual studies on suicidality and self-harm. Based on the data synthesis, it compared the public health impact of exposure on the population of the identified exposure. Bullying victimization was the most attributed environmental exposure for suicidality. The other identified significant school and individual factors were sleeping disturbance, school absenteeism, and exposure to antidepressants. Several significant vulnerable young populations were identified with significantly higher prevalence of suicidality, including lesbian, gay, bisexual, transgender, queer (or questioning) youth and those with mental health disorders, problem behaviors, previous suicidality, self-harm, and gender (female). A person-centered approach emphasizing connectedness and bully-free school environments should be a priority focus for schools, health professionals, and public health policymakers.

## Introduction

Suicide remains one of the most common causes of death in adolescents, defined as those between the ages of 10 and 19 years (WHO, 2023), and is a growing concern globally (Hawton & Harriss, 2007; Patton et al., 2009; UNICEF, 2021). Self-harm (without suicide intent) in adolescence is also a widespread issue, with the prevalence rate of repetitive self-harm being around 20% (Xiao et al., 2022). In young people, suicidal behavior and self-harm are more common than suicide deaths but are associated with other negative consequences such as co-morbid mental health issues and impact on education and work (Cox & Hetrick, 2017). Even though suicidality and self-harm are two distinctively defined mental health outcomes, they share common risk factors (Figueiredo et al., 2023; Ougrin, 2014). The current literature provides a large volume of individual studies reporting factors associated with these outcomes. There is limited research focused on identifying and understanding suicidality and self-harm risk and protective factors as a whole for school students. This study used umbrella review and meta-analysis to synthesize review evidence for these outcomes and identify risk and protective factors, particularly for school students.

Analysis of school-based risk factors is essential, given that adolescents spend most of their time at school (Surgenor et al., 2016). Due to COVID-19, many countries decreased school attendance due to pandemic-related medical absences and adherence to lockdown advice. School absenteeism has become a more severe education and public health issue, and this is concerning given previous identification of school absenteeism as a significant factor associated with suicidality and self-harm (Aggarwal et al., 2017). This umbrella review will provide a narrative synthesis focusing on school-based factors.

Existing literature identified various contextual risk factors that may increase the likelihood of self-harm and suicidal behavior in adolescents, including individual, relational, community, or societal factors (Bilsen, 2018; Fergusson et al., 2000; Kennebeck et al., 2017). Commonly reported individual factors include previous experiences of mental health issues, suicidal behaviors, and other forms of injury and violence (CDCP, 2022). Common examples of relationship factors include family and childhood experiences, relationship breakdowns, social isolation, and bullying (CDCP, 2022). Community and societal risk factors, acting as the environmental influence, include barriers to accessing healthcare, cultural beliefs, stigma attached to mental health illness(es) or sexual orientation, and ease of access to dangerous items or other means of self-harm (CDCP, 2022).

Considering the multilevel features of these contextual risk factors, globally adopted public health and education strategies are to allocate resources to eliminate exposed risk from these factors (Dragioti et al., 2022). Under a resource-constrained environment with numerous complex risk factors, estimating the quantified attributable impacts of these risk factors on mental health outcomes will help derive targeted preventive programs. Using Meta-analysis from the umbrella review will potentially estimate the relative attributable implications of the identified factors.

There are also protective factors of suicidality and self-harm in the resiliency framework to inform intervention and prevention (Zimmerman et al., 2013). Common examples of protective factors against suicidality and self-harm include connectedness, supported relationships, and healthcare access (AAP, 2022; CDCP, 2022). However, less is understood about how protective factors promote resilience to adolescent suicidality (Gallagher & Miller, 2018). This review will search the review literature for protective factors and explore their shared effects on suicidality and self-harm. Nevertheless, there is no consensus on the definition of protective factors. Wright et al. (2013) described the protective process as including ”protective factors” and “compensatory factors.” For the current review, protective factors are those directly associated with the lower probability of outcomes, i.e., the promotive factor/direct protective factor (Lösel & Farrington, 2012). This definition is more in line with the review by Gubbels et al. (2023), which defined protective factors as those directly associated with adverse outcomes as opposed to moderating the risk factors of these outcomes.

## Current study

As indicated above, a plethora of studies are being published on the individual risk and protective factors in adolescents. Using a synthesis method to compare findings from the synthesized evidence (i.e., existing reviews and meta-analysis) offers an efficient way to help researchers gain a broader perspective from the large scales of evidence. Synthesis methods can help to clarify the strength and consistency of findings and highlight areas where more research may be needed. Umbrella reviews adopt explicit and systematic methods to search and identify systematic reviews and meta-analyses, which is helpful for this purpose. This study aims to use an umbrella review method (systematic review of systematic reviews) to synthesize reviewed studies about adolescent suicidality and self-harm, their risk, and protective factors. The second aim is to identify, summarize, and quantify any findings relating to individual school factors (including absenteeism) from the review literature. The research question is: What are the main reported risk and protective factors of self-harm and suicidality in adolescents in the review literature?

## Methods

### Umbrella Review

This study used an umbrella review method to synthesize evidence from the published literature over the past 20 years about the risk and protective factors of self-harm and suicidality in adolescents. This umbrella review only included results from the quantitative synthesis of systematic reviews/narrative reviews/meta-analyses as the unit of searching, inclusion, and analysis. The Joanna Briggs Institute’s evidence-based healthcare article (Aromataris et al., 2015) and other methodology guidelines were used to guide/inform the methodology, data extraction, and quality appraisals. A checklist was developed according to Assessing the Methodological Quality of Systematic Reviews to assess the quality and bias of each systematic review/meta-analysis. A data collection tool was generated to record the studies identified. Preferred Reporting Items for Overview of Reviews (Pollock et al., 2019) was used as the guideline for umbrella review reporting. Tabulation summaries and narrative synthesis were used to compare findings from existing quantitative meta-analysis studies.

### Types of participants and studies

Adolescents were defined as individuals aged 10 to 19 (WHO, 2023), also referred to as teenagers. Due to various age definitions of adolescents in the quantitative reviews, a wider age range (9–25) was included. The study included reviews for adolescents and children; however, only data relevant to adolescents were extrapolated. The types of studies included systematic review and meta-analysis of RCT or observational studies and review of qualitative studies, including narrative reviews. The results presented in the current study were limited to systematic reviews with meta-analyses from interventional and observational quantitative meta-analyses.

### Context/setting

As a New Zealand study of global literature, this review included current grey literature in the search from Māori and Pacific publications as well as New Zealand government documentation and research hosted on government or organizational websites (e.g., Le Va, Te Pou, Nga Pae o Te Maramatanga, Te Rau Ora, and Whakauae).

### Outcomes

The study outcomes were quantitative measures of mental health outcomes with known shared exposures in adolescent’s developmental life. They specifically included suicidality, which encompassed suicidal behavior (SB), defined as intentional action on self to cause one’s death, and suicidal ideation (SI), regarded as thoughts of action to end one’s own life with no intent to act. The other outcome was self-harm, defined as the deliberate act of hurting one’s own body without suicide intent. Self-harm may include cutting skin, biting, burning, or scratching skin, head banging or hitting oneself, and taking overdoses or harmful substances (Mental-Health-Foundation-New-Zealand, 2022). Although there are different definitions of self-harm (e.g., deliberate self-harm, self-injury, Non-Suicidal Self Injury), the current review used the term self-harm to refer to any intentional injury to oneself without suicidal intention as described in the DSM-5 (Figueiredo et al., 2023), self-harm with suicidal intent was included under suicidality. Articles included in this review have addressed either suicidality, self-harm, or both.

### Search Strategy

**Fig. 1:**
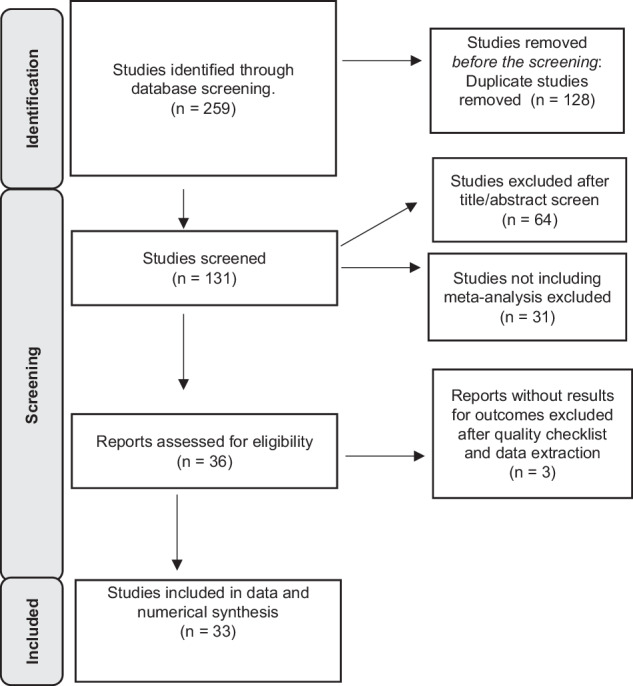
Prisma flowchart

The search included published systematic reviews and meta-analysis journal articles written in English and published from 2003 until 30^th^ Dec 2022. Search databases included Medline, PubMed, Embase, CINAHL, and PsycINFO. The keywords and filter used in the initial search were: [teen* OR adolescent* OR youth OR “young people”] (Title/Abstract) AND [suicid* OR self-harm* OR self-inju*] (Title/Abstract) AND [risk* OR predispos* OR cause* OR protect* OR prevent*] (Title/Abstract) AND [“Systematic review” OR meta-analysis] (Title/Abstract). Each set of keywords was searched in the abstract and title fields. Medical subject headings (MeSH terms) were not used. A hand search from reference lists was undertaken for articles discussing antidepressants and other factors. The relevance of reviews was assessed through the title, abstract, and subject terms/index terms/keywords. Two reviewers (RFR, I.Z.) conducted separate searches under the guidance of two senior librarians, with identified articles merged for screening. The screening was conducted independently based on the relevance of the review articles’ abstracts, keywords, methods, and outcomes. A final consensus was reached through discussions. The review searching process was summarized using a PRISMA flowchart in Fig. 1.

### Inclusion and exclusion Criteria

**Fig. 2:**
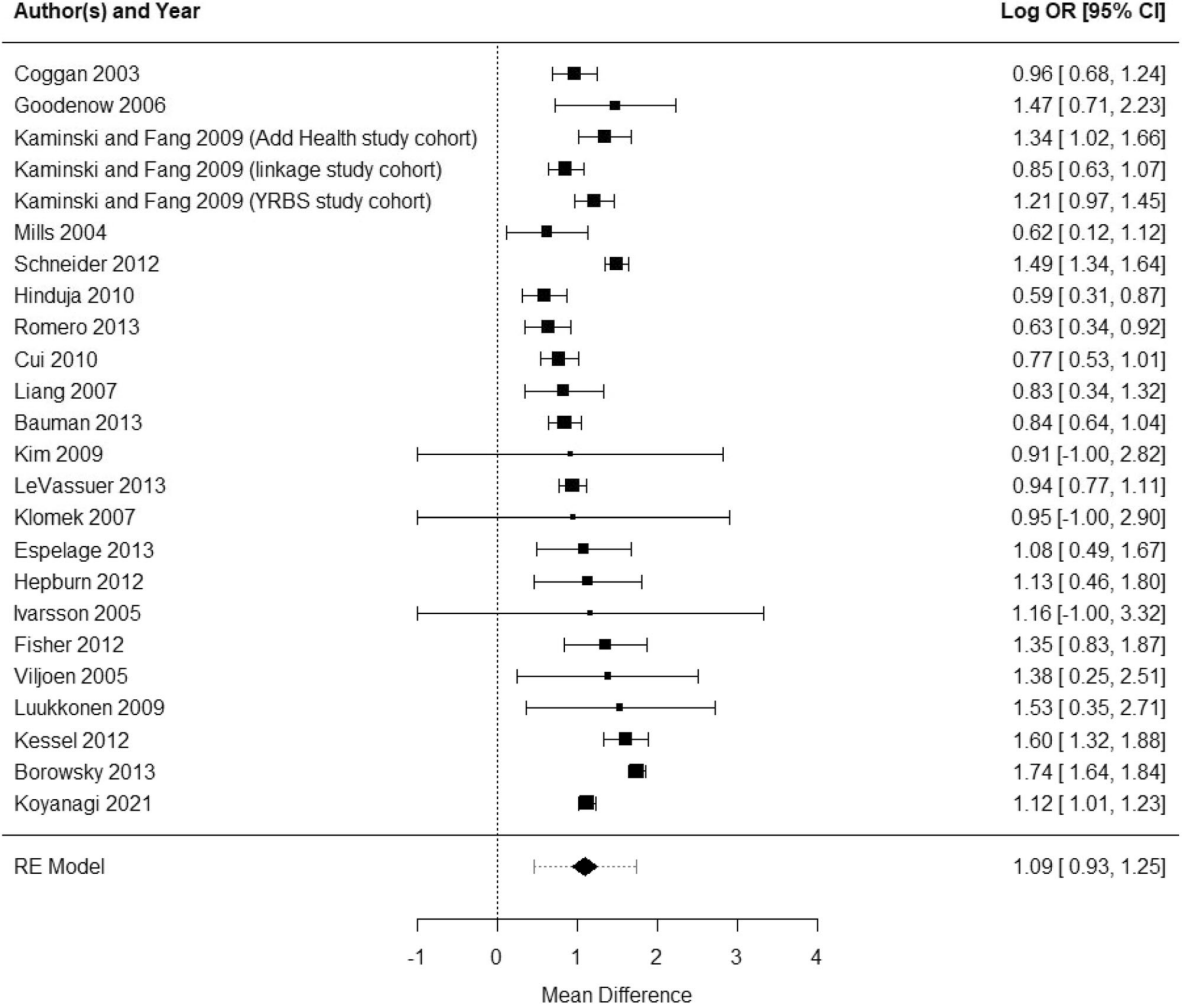
Forest plot

**Fig. 3:**
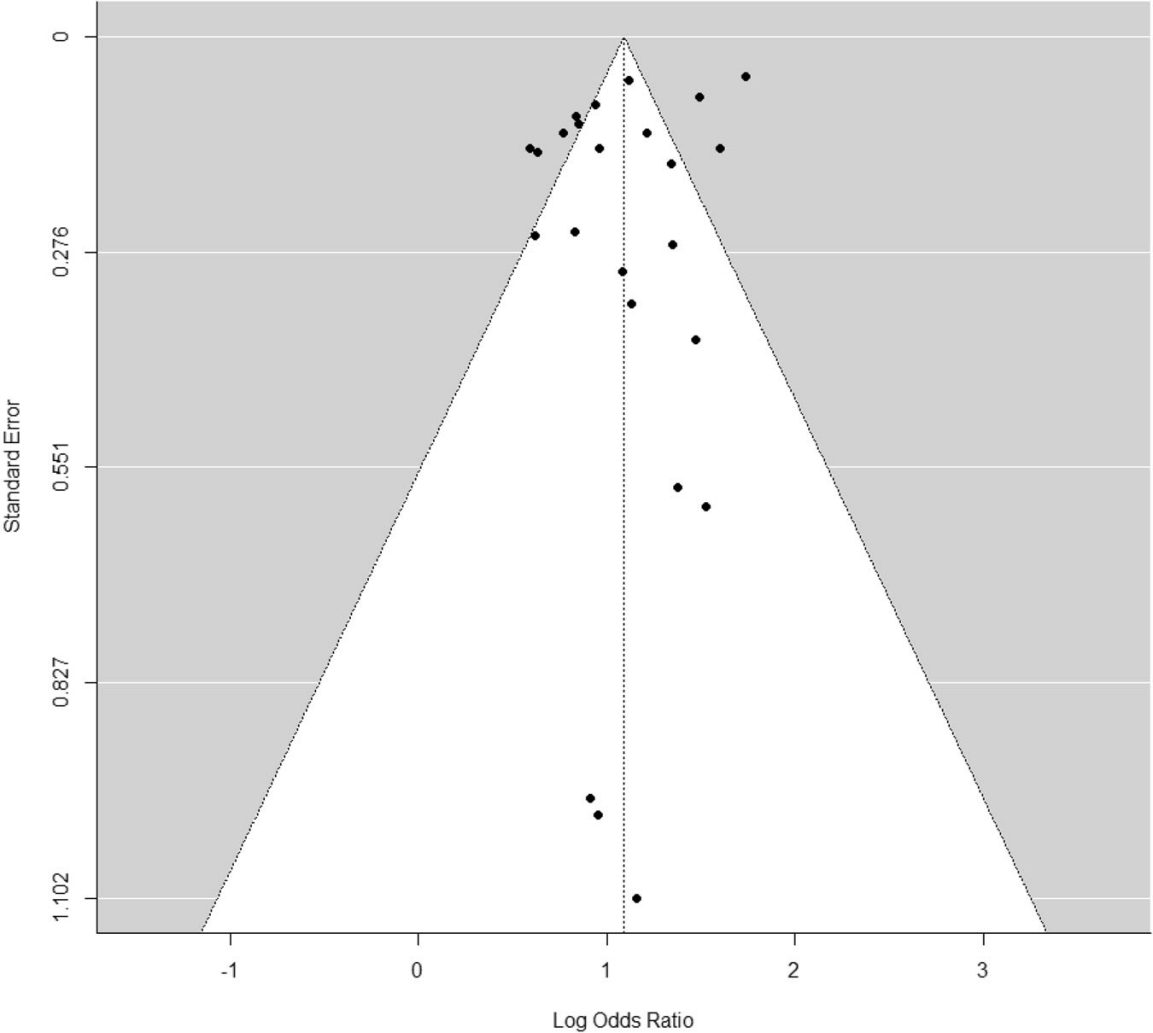
Funnel plot

The umbrella review utilized the PICOS (participants, intervention, comparators, outcomes, and study design) structure to decide on its inclusion and exclusion criteria. Articles included in this review addressed risk factors, protective factors (factors mitigating risk factors), or both for adolescents’ self-harm and suicidal behavior. The included risk factors, which predispose an individual to specific mental health outcomes, can be at an individual, relational, community, or societal level. The review excluded protocols, guidelines, letters from editors, reviews of other mental health outcomes, reviews of other age groups, non-English reviews, and reviews not including meta-analysis (Figs. 2, 3).

### Methodological Quality and Epidemiologic Credibility Assessment

The methodological quality of the retrieved studies was assessed using the Joanna Briggs Institute (JBI) “Critical Appraisal Checklist for Systematic Reviews and Research Synthesis” (Aromataris et al., 2015). Each question on the checklist was coded as being 1 (yes), 0 (no), or N/A (not applicable). A total methodological quality score was calculated by adding the items scored as yes for each review. The screening was conducted independently by two reviewers (IZ, TC), and consensus was reached through discussion by three reviewers (IZ, TC, RFR). Microsoft Excel was used for data extraction, screening, and quality assessment (Appendix 1). The epidemiologic credibility of the risk and preventative factors were assessed using the criteria established in a previous umbrella review (Köhler et al., 2018). The findings were rated independently by three reviewers (IZ, TC, RFR) as I (convincing evidence), II (highly suggestive evidence), III (suggestive evidence), IV (weak evidence), N/A (not applicable), and NS (non-significant findings).

### Numerical synthesis

The exposure risk factors with sufficient data in the extractions and relevant to school life, including school absenteeism, bully victimization, and sleep disturbance, were analyzed to derive their Population Attributable Fraction (PAF) (Mansournia & Altman, 2018). PAF measures the public health impact of exposure on the population. It represents the proportion of adverse outcomes/cases that would not have occurred without exposure. PAF is determined by the prevalence of the exposure and the strength of the association between the exposure and the adverse outcome. If exposure has a larger PAF than the others, it will indicate its more significant attributable fraction to the adverse outcome.

The prevalence of exposures (bully victimization, sleep disturbances) was obtained from the WHO-initiated Global School Health Survey (GSHS) (WHO, 2023), which was used to derive PAF.

The extracted prevalence rates of bully victimization and sleep disturbances from the GSHS were reported by Biswas et al. (2020a, 2020b) and Hasan et al. (2023), respectively. Prevalence rates of school absenteeism were obtained from a meta-analysis of global studies (Gubbels et al., 2019) and New Zealand’s latest government registry of school attendance (EducationCounts, 2023). The pooled Odds Ratios (OR) of exposures to outcomes were extracted from the review and meta-analysis studies (Epstein et al., 2020; Holt et al., 2015; Koyanagi et al., 2019; Liu et al., 2019; Muehlenkamp et al., 2012; Van Geel et al., 2014). The Risk Ratios (RR) were approximated from the reported OR (Zhang & Yu, 1998) using an estimated prevalence (p_0_) of the outcomes in the general population (as for the control group), given by meta-analysis studies (Lim et al., 2019) and the GSHS global population surveys (Biswas et al., 2020a, 2020b). The geometric mean of the inversed variance weighted log (OR) from two meta-analysis studies (Holt et al., 2015; Van Geel et al., 2014) was used to derive a population estimate (OR) for bully victimization. PAF was then calculated using the Bayes formula (Fergusson et al., 2000; Lin & Chen, 2019; ML, 1953). Two different PAFs were derived; one estimate used the OR to approximate RR, and the other used the converted RR. The permuted distributions of PAF were generated through permutations of OR and exposure prevalence to derive PAF’s 95% confidence intervals (CI).

Pooling all individual studies from meta-analyses (Holt et al., 2015; Koyanagi et al., 2019; Van Geel et al., 2014), an umbrella review meta-analysis was conducted to examine the association between bullying victimization (including victimization from peer bullying and other bullying) and recent suicide attempts. Duplicated studies were removed. Included studies have used the effect size measured by variables obtained at the same time point (from cross-sectional studies or the cross-sectional part of a longitudinal study). The random effect model was used due to the significant study heterogeneities. OR were natural log-transformed, and the variance was estimated from each study. The degree of between-study heterogeneity was assessed using I^2^ statistics. Sensitivity analyses were conducted to evaluate each study’s impact on the pooled OR. Publication bias was inspected by funnel plot and Egger’s regression test for asymmetry. A significant Egger’s test statistic (*p* < 0.05) suggests substantial asymmetry in the funnel plot, which may indicate publication bias. Moderator analyses were conducted to ascertain if sample characteristics (years of publication and country) impacted the effect size estimate. R package “Metafor” (Viechtbauer, 2023) was used for the meta-analysis.

### Registration

Before the review and data extraction, the study was registered at PROSPERO, an international database of prospectively registered systematic reviews in health and social care (study number CRD42023392414).

## Results

The current study included the results from a quantitative synthesis of 33 systematic reviews with meta-analysis (Fig. 1); these reviews included 1149 individual studies, with a minimum of three studies and a maximum of 369 studies. The individual studies (cross-sectional, case-control, cohort, and randomized control trials) included community and clinical samples. The data that support the findings of this study are available from the repository: https://github.com/suicideprevention/umbrella-review-data-for-synthesis.

### Outcome Measures for Suicidal Behavior, ideation, and self-harm

Suicidal behavior is defined as actions with the intention to cause oneself to die. Text Revised Fifth Edition of the Diagnostic and Statistical Manual of Mental Disorders (DSM-5-TR, 2022) defines it as the engagement in self-directed injurious behavior with the intent to die. Suicidal behavior in these reviews was measured predominantly through self-report or peer-report questionnaires and structured interviews. These reviews included both standardized and non-standardized measures, such as the Beck Depression Inventory, Youth Risk Behavior Surveillance System, Youth Self-Report, Moods and Feelings Questionnaire, Kiddie Schedule for Affective Disorders and Schizophrenia, and the Adolescent Suicide interview. Most reviews included two or more items regarding suicidal behaviors. However, some reviews included studies with only one item addressing suicidal behaviors. For reviews on antidepressant use, suicidal behavior was measured through recorded adverse events, ICD-9 or ICD-10 codes, and medical records. Most reviews explored past suicidal behavior (often six or 12 months), but some also explored the risk of current and future behaviors. The results for youth suicidal behavior are presented in Table 1.

**Table 1:** Youth Suicidal Behavior

Total reviews included	Study Population	Risk Factors/ Protective Factors	Findings	Study Population (Total reviews included)	Risk Factors/ Protective Factors	Findings
23	9–26 years, Includes vulnerable groups (youth with social anxiety, high-risk students, Sexual minority youth, youth with depressive disorder)	Bullying/ peer victimization (Holt et al., 2015; Koyanagi et al., 2019; Van Geel et al., 2014)	OR range: 2.78–3.26	School-aged students, including high-risk and sexual minority students. (5 reviews)	Bullying victimization (Koyanagi et al., 2019)	OR: 3.06
		Bullying Perpetration (21)	OR: 2.62			
		Antidepressant use (Barbui et al., 2009; Dubicka et al., 2010; Hetrick et al., 2012; Li et al., 2022)	OR range: 1.58–1.92 RR range: 1.26–1.35			
		Female Gender (Miranda-Mendizabal et al., 2019; Van Meter et al., 2022)	OR: 1.96 8.5% prevalence of SB			
		Sexual orientation (Marshal et al., 2011; Miranda-Mendizábal et al., 2017)	*OR range: 2.26–2.92 *Bisexuality OR: 4.92			
		Previous self-harm (Castellví et al., 2017; Gillies et al., 2018)	OR: 2.26 RR: 9.14		Previous self-harm (Gillies et al., 2018)	RR: 9.14
		Previous suicidal ideation (Castellví et al., 2017; Miranda-Mendizabal et al., 2019)	OR: 3.26 Female OR: 4.39, Male OR: 3.97			
		Previous suicide attempt (Castellví et al., 2017; Miranda-Mendizabal et al., 2019)	OR: 5.56 Female OR: 6.96			
		Previous suicidal thoughts and behaviors (Castellví et al., 2017)	OR: 3.48			
		Any mental health disorder (Gili et al., 2019; Miranda-Mendizabal et al., 2019)	OR: 3.57 Female OR: 3.37, Male OR: 4.23			
		Depression symptoms (Miranda-Mendizabal et al., 2019; Soto‐Sanz et al., 2019)	OR: 6.57 Female OR: 1.15			
		Bipolar disorder (Hauser et al., 2013)	Prevalence Past:1.3% Current:25.5%			
		Anxiety disorder (Miranda-Mendizabal et al., 2019)	Female OR: 2.03 Male OR: 3.79			
		Major Depressive disorder(Miranda-Mendizabal et al., 2019)	Female OR: 4.49 Male OR: 6.07			
		Social anxiety (Leigh et al., 2023)(9)	r range: 0.1–0.24			
		Cannabis use (Gobbi et al., 2019)	OR: 3.46			
		Sleep disturbances (Liu et al., 2019)	OR: 1.92		Sleep disturbances (Liu et al., 2019)	OR: 1.92
		Autism (O’halloran et al., 2022)	8.3% prevalence			
		Childhood maltreatment (Miranda-Mendizabal et al., 2019)	Female OR: 3.77 Male OR: 2.76			
		Sleep duration (Chiu et al., 2018)	OR: 0.52		Sleep duration (Chiu et al., 2018)	OR: 0.52
		General internalizing symptoms (Soto‐Sanz et al., 2019)	ES: 0.93			
		Externalizing symptoms (Soto‐Sanz et al., 2019)	ES: 0.93 OR: 2.59			
		Legal problems (Soto‐Sanz et al., 2019)	OR: 3.36			
		School connectedness(Marraccini & Brier, 2017)	OR: 0.59 High-risk youth OR: 0.60 Sexual minority youth OR: 0.61		School Connectedness (Marraccini & Brier, 2017)	OR: 0.59 High-risk youth OR: 0.60 Sexual minority youth OR: 0.61
		Prevention of STBs at school (Gijzen et al., 2022)(23)	Hedges’ g: 0.3			

*OR* pooled odds ratio, *RR* pooled risk ratio

^*^Marshal et al. (2011) combined suicide behavior and ideation as outcomes in their meta-analysis

Suicidal ideation is defined as thinking about (thoughts) or planning suicide. Suicidal ideation in these reviews was measured through many ways, such as self-report and peer-report standardized tools, non-standardized interviews/questions, or official records. Examples of measures used included the Beck Scale for Suicide Ideation, the Schedule for Affective Disorders and Schizophrenia for School-Aged Children, the Youth Risk Behavior Surveillance System, the Kiddie Schedule for Affective Disorders and Schizophrenia, and the Paykel Hierarchical Ladder of Suicide. For reviews of antidepressant use, suicidal ideation was measured through suicide-related events reported by the Committee on Safety Medicines and Treatment for Adolescents with Depression Study and individual events (Dubicka et al., 2010; Hetrick et al., 2012). Most reviews explored past suicidal ideation (often 6 or 12 months). The results for youth suicidal ideation were presented in Table 2.

**Table 2:** Youth Suicidal Ideation

Total reviews included	Study Population	Interventions- Risk/ Protective Factors (included meta-analyses)	Findings	Study Population (Total reviews included)	Interventions - Risk/ Protective Factors (included meta-analyses)	Findings of the meta-analysis
19	9–23 years	Female gender (McKinnon et al., 2016; Van Meter et al., 2022)	prevalence range: 11.4%–19.8% RR range: 1.19–1.24	School-aged students, including high-risk and sexual minority students. (8 reviews)	Female Gender (McKinnon et al., 2016)	16.2% prevalence RR ranges from 1.19–1.24
		Antidepressant use (Dubicka et al., 2010; Hetrick et al., 2012)	OR range: 1.58–1.63			
		Bipolar disorder (Das Neves Peixoto et al., 2017; Hauser et al., 2013)	RR: 2.94 r: 50.4–57.4			
		Bullying/peer victimization (Holt et al., 2015; Van Geel et al., 2014)	OR range: 2.12–2.34			
		antidepressant use (Dubicka et al., 2010; Hetrick et al., 2012)	OR range: 1.58–1.63			
		Previous self-harm (Gillies et al., 2018)	RR: 4.97		Previous self-harm (Gillies et al., 2018)	RR: 4.97
		Social anxiety (Leigh et al., 2023)	r: 0.22			
		Cannabis use (Gobbi et al., 2019)	OR: 1.5			
		Sleep disturbances (Liu et al., 2019)	OR: 2.35 With plan OR: 1.58		Sleep disturbances (Liu et al., 2019)	OR: 2.35 With plan OR: 1.58
		Autism (O’halloran et al., 2022)	25.2% prevalence			
		Cyber-bullying victimization (Kowalski et al., 2014)	r: 0.27		Cyber-bullying victimization (Kowalski et al., 2014)	r + : 0.27
		Sexual orientation (Marshal et al., 2011)	*OR: 2.92 *Bisexuality OR: 4.92			
		School absenteeism (Epstein et al., 2020)	OR: 1.2		School absenteeism (Epstein et al., 2020)	OR: 1.2
		School connectedness (Marraccini & Brier, 2017)	OR: 0.53		School Connectedness (Marraccini & Brier, 2017)	OR: 0.53
		Prevention of STBs at schools (Gijzen et al., 2022)	Hedges’ g: 0.15		Prevention of STBs at schools (Gijzen et al., 2022)	Hedges’ g: 0.15
		Sleep duration(Chiu et al., 2018)	OR: 0.55 With plan OR: 0.50		Sleep duration (Chiu et al., 2018)	OR: 0.55 With plan OR: 0.50

*OR* pooled odds ratio, *RR* pooled risk ratio

^*^Marshal et al. (2011) combined suicide behavior and ideation as outcomes in their meta-analysis

Self-harm is defined as deliberately harming oneself without intent to die and is also referred to as non-suicidal self-injury (NSSI; DSM-5). Self-harm was measured through both standardized and non-standardized self-report, interviews, questionnaires, and official reports. Examples of measures used included the Risky Behavior Questionnaire for Adolescents, Self-injury Questionnaire-treatment related, Functional Assessment of Self-mutilation, Ottawa self-injury, Adolescent NSSI behavior questionnaire, and Deliberate Self-harm Inventory. The included reviews explored past self-harm, focusing on the past 6 or 12 months (Table 3).

**Table 3:** Youth Self-Harm

Total reviews included	Study Population	Interventions- Risk/ Protective Factors (Included Meta-Analyses)	Findings	Study Population (Total reviews included)	Interventions - Risk/ Protective Factors (included meta-analyses)	Findings of the meta-analysis
10	10–25 years, Includes vulnerable groups (Youth with depressive disorder, Autistic Youth)	Female gender (Gillies et al., 2018; Wang et al., 2022; Xiao et al., 2022)(8, 25, 33)	RR: 1.72, OR: 2.89 25.4% prevalence	School-aged students, including high-risk and sexual minority students. (4 reviews)	Female Gender (Gillies et al., 2018; Xiao et al., 2022)	RR: 1.72 Prevalence rate: 25.4
		Bullying victimization (Heerde & Hemphill, 2019; Wang et al., 2022)	OR ranges from 1.98–2.34		Bullying victimization (Heerde & Hemphill, 2019)	OR: 2.34
		Antidepressant use	OR: 1.44			
		Bullying perpetration (Heerde & Hemphill, 2019)	OR: 1.81		Bullying perpetration (Heerde & Hemphill, 2019)	OR: 1.81
		Cyber-bullying victimization (Heerde & Hemphill, 2019)	OR: 3.55		Cyber-bullying victimization (Heerde & Hemphill, 2019)	OR: 3.55
		Autism (O’halloran et al., 2022)	8.3% Prevalence (suicide attempt/self-harm)			
		Smoking history (Xiao et al., 2022)	24.7% Prevalence		Smoking history (Xiao et al., 2022)	24.7% Prevalence
		Alcohol history (Xiao et al., 2022)	24.4% Prevalence		Alcohol history (Xiao et al., 2022)	24.4% Prevalence
		Family with multiple children (Xiao et al., 2022)	27% Prevalence		Family with multiple children (Xiao et al., 2022)	27% Prevalence
		Single-parent families (Xiao et al., 2022)	30.1% Prevalence		Single-parent families (Xiao et al., 2022)	30.1% Prevalence
		School absenteeism (Epstein et al., 2020)	OR: 1.37		School absenteeism (Epstein et al., 2020)	OR:1.37
		Adverse childhood experiences (Wang et al., 2022)	OR: 2.49			
		Low health literacy (Wang et al., 2022)	OR: 2.2			
		Mental disorders (Wang et al., 2022)	OR: 1.89			
		Physical symptoms (Wang et al., 2022)	OR: 2.85			
		Problem behaviors (Wang et al., 2022)	OR: 2.36			

*OR* pooled odds ratio, *RR* pooled risk ratio

### Narrative Data Synthesis

#### Exposures risk factors

##### Antidepressants

The most reviewed risk factor for suicidal behavior in the literature was antidepressants, including selective serotonin reuptake inhibitor (SSRI) and the new generation of antidepressant exposure. These studies focused on youth aged between 9–25 years, including participants diagnosed with a depressive disorder. Antidepressant use was measured through randomized control trials and observational studies of Mirtazapine, Fluoxetine, Paroxetine, Sertraline, Citalopram, Fluvoxamine, Venlafaxine, and Escitalopram. In the included randomized control trials (Dubicka et al., 2010; Hetrick et al., 2012), the pooled OR were derived from comparisons between the antidepressant and placebo groups. The suicide behaviors were reported as suicide-related outcomes or adverse events in these studies, including suicide attempts, completed suicide, and suicidal self-harm. In the observational studies (Barbui et al., 2009; Li et al., 2022), antidepressant intake was included as an exposure in cohort and case-control studies. Barbui et al. (2009) found that the OR for the overall relationship between SSRI and suicidal behavior was 1.92 (95% CI, 1.51–2.44). Dubicka et al. (2010) found the OR for antidepressant exposure was 1.70 (95% CI, 1.13–2.54). Hetrick et al. (2012) examined the association between antidepressant use and the combined outcomes of suicidal behavior and ideation, resulting in a pooled RR of 1.58 (95% CI 1.02- 2.45). The latest review on this topic by Li et al. (2022) featured the most significant number of individual participants from 11 studies and found that the pooled RR of antidepressant exposure (including SSRI) was 1.38 (95% CI: 1.16–1.64), and for SSRI exposure only was 1.28 (95% CI: 1.09–1.51). The result indicated that the risk of suicidal behavior in youth exposed to antidepressants was 38% higher (Table 1).

Dubicka et al. (2010) found suicidal thoughts, which occurred in nine of 738 (1.2%) young people with depression treated with antidepressants compared to five in 634 of those treated with a placebo (2.6%). The results indicated a significant association between the risk of youth suicidal thoughts and antidepressant use (OR*:*1.45). Both Dubicka et al. (2010) and Hetrick et al. (2012) explored the combined outcomes of suicidal behavior and ideation (Table 2).

Dubicka et al. (2010) also reported that self-harm occurred in 19 of 569 (3.3%) young people with depression treated with antidepressants compared to 12 in 469 of those treated with a placebo (2.6%). The results represented a significant association between the risk of youth self-harm and antidepressant use, with an OR of 1.44 (Table 3).

Despite the identified reviews being from different types of studies (i.e., RCT vs. observational) and the corresponding pooled effect size using OR (cross-sectional) or RR (cohort), their results in antidepressants and suicidal behavior are in the similar range between 1.70 and 1.92, which are higher than the effect size of suicidal ideation. The same exposure also had a similar effect size in self-harm- a different outcome from suicide ideation. Review studies of RCT have not explicitly reported results for subgroup populations (different genders or age groups) and included both clinical samples and samples from communities. The observational review (Barbui et al., 2009), including different age groups, suggested a promoting effect of SSRI exposure among adolescents but a protective effect in adults and the elderly. The data synthesis from the current umbrella review, including the recent observational review (Li et al., 2022), suggested that the increased risk of suicidal behavior for SSRIs and other antidepressants was similar in both children and adolescents.

##### Bullying Victimization and Perpetration

Three studies explored the relationship between bullying/peer victimization and suicidal behavior (Holt et al., 2015; Koyanagi et al., 2019; Van Geel et al., 2014). Koyanagi et al. (2019) included studies of the GSHS from 48 low and middle-income countries; the other two reviews included participants aged between 9–23 years from low-, middle-, and high-income countries. These reviews included studies that measured bullying according to the Centers for Disease Control and Prevention’s uniform definition and included the following components: providing a definition of bullying/peer victimization followed by questions, measuring aggressive acts, measuring power imbalance or differential, or direct asking if the participant was bullied. The OR ranged from 2.78 to 3.26 for the overall relationship between bullying victimization and suicidal behavior. Holt et al. (2015) reviewed 15 studies that assessed the relationship between bullying perpetration and suicidal behavior, measured through behaviorally based questions and directly asking students if they had bullied others. They found a significant OR of 2.62 (95% CI, 1.51–4.55), indicating a 1.62-fold higher risk of suicidal behavior for youth who perpetrated bullying (Table 1).

Holt et al. (2015) and Van Geel et al. (2014) explored bullying victimization as a risk for suicidal ideation from 23 and 24 individual studies, respectively; the pooled OR was 2.23 and 2.34 for the overall relationship between bullying victimization and suicide ideation. These results indicated a 1.2–1.3-fold higher risk of suicidal ideation for youth exposed to bullying. Holt et al. (2015) reviewed studies that assessed the relationship between bullying perpetration and suicidal ideation and found a pooled OR of 2.12. Kowalski et al. (2014) investigated cyberbullying specifically. They found that youth who reported high levels of cyberbullying victimization were also likely to report high levels of suicidal ideation with a correlation coefficient (*r*: 0.27) (Table 2).

Heerde and Hemphill (2019) and Wang et al. (2022) reviewed the association between general bullying victimization (both traditional and cyberbullying) and youth self-harm from three and seven studies, respectively. The two reviews found that OR ranged from 1.98 to 2.34 for the relationship between bullying victimization and youth self-harm. Heerde and Hemphill (2019) explored traditional bullying and cyberbullying separately; they reported the association between cyberbullying victimization and risk of self-harm with a pooled OR of 3.55 (Table 3).

The synthesis of these reviews and meta-analysis identified both bullying victims and perpetration as common risk factors for suicide behavior, ideation, and self-harm. Risk is higher in suicidal behavior than ideation from bully victimization, and there is a similarity between suicidal ideation and self-harm. The cross-review comparison suggested that bully victimization has a slightly higher risk of suicide behavior and ideation compared to bully penetration; cyberbullying has a higher risk than traditional bullying in self-harm. All reviews included samples from schools and communities; one included mental health clinics and another setting. Most studies included in these reviews are cross-sectional, with a few longitudinal design studies included (Holt et al., 2015).

One review (Holt et al., 2015) found a significant moderating effect from the country of origin of the study in both bullying perpetration and victimization. Gender was reported in individual studies as a moderator to associations between bullying and suicidality (Klomek et al., 2009) but not significant in Holt’s meta-analysis. The other moderators with non-significant results included the sampling methods (cluster, stratified, simple random sample or census) and measurements of bullying (single vs multiple) (Van Geel et al., 2014). The meta-analysis of Koyanagi et al. (2019) also tested different forms of bullying in the order of their effect sizes, which are religion, race/nationality/color, physical bullying, sexual bullying, exclusion, and being made fun of for physical appearance.

##### Sleep disturbance

Liu et al. (2019) conducted a review including 34,933 participants to explore sleep disturbances defined as difficulty sleeping, insomnia symptoms, and poor sleep, measured by various standardized scales and questionnaires. Their review included ten cross-sectional and four longitudinal studies and found that sleep disturbances were associated with a higher risk of adolescent suicide attempts (OR: 1.92) and a higher risk of adolescent suicidal ideation (OR: 2.35) as well as suicidal ideation with a plan (OR: 1.58). The significant moderators on the association between sleep disturbances and suicide ideation were using insomnia symptoms in measurement, age, and reliable sleep measures. The female percentage in studies was a significant positive moderator on the association for suicide attempts.

#### Vulnerable populations

##### Female

Miranda-Mendizabal et al. (2019) and Van Meter et al. (2022) all identified that suicidal attempts were more prevalent in female youth than male youth. These two reviews comprised youth from multiple countries. Miranda-Mendizabal et al. (2019) found that female youth were almost twice as likely to experience suicidal behavior (OR: 1.96). Van Meter et al. (2022) found an 8.5% prevalence in female youth reporting suicidal attempts compared to a 4.9% prevalence in males (Table 1). In addition, both reviews found that suicidal ideation with a plan was higher in female youth than male youth, with the pooled prevalence for females ranging from 11.4 to 19.8% (Table 2). The most reported risk factor for self-harm was gender; Gillies et al. (2018), Xiao et al. (2022), and Wang et al. (2022), all identified that the risk of self-harm was more prevalent in female youth compared to male youth. Gillies et al. (2018) reviewed 261 studies, including 597,548 participants, and reported that the female gender had a pooled RR of 1.72. In a review of 43 studies with 107,285 male and 102,473 female participants, Xiao et al. (2022) found a 25.4% NSSI prevalence for female adolescents compared to a 22% prevalence for males. In a review of 8 studies, Wang et al. (2022) found a significant association between female gender and a significant risk of self-harm (OR*:* 2.89) (Table 3).

##### LGBT

Miranda-Mendizábal et al. (2017) studied the association between sexual orientation and youth suicidal behavior, including six studies with 22,117 participants. The pooled OR for the overall relationship between sexual orientation and youth suicidal behavior is 2.26. Marshal et al. (2011) studied the association between sexual orientation and youth suicidality (behavior and suicidal ideation) through 19 studies with 122,955 participants, revealing a pooled OR of 2.92. The review also investigated specifically bisexual youth, including four studies with 42,413 participants, and found a significant association between bisexual youth and suicidal behaviors and ideations (OR*:* 4.92) (Tables 1, 2).

##### Adolescents with Mental Health Disorders

Six reviews explored the associations between various mental health disorders (including anxiety disorders and major depressive disorders) and suicidal behavior. Gili et al. (2019) found that any presence of mental health disorders increased the risk of suicidal attempts (OR: 3.57). Miranda-Mendizabal et al. (2019) investigated mental health disorders and suicidal behavior with male and female gender separately. The mental health issues associated with female suicidal behavior included anxiety disorder, drug abuse disorder, major depressive disorder, depressive symptoms, and any mental disorder or abuse. The OR ranged from 1.15 to 4.49 for the overall relationship between mental health disorders and female suicidal behavior. Mental health issues associated with male suicidal behavior identified in this review included anxiety disorder, major depressive disorder, and any mental disorder or abuse, with the OR ranging from 3.79 to 6.07. Major Depressive Disorder had the highest association with suicidal behavior for female youth (OR: 4.49) and male youth (OR: 6.07) (Table 1). Hauser et al. (2013) reviewed 11 studies that specifically explored the link between bipolar disorder and suicidality. Findings from the review showed a 21.3% prevalence of past suicide attempts and a 25.5% prevalence of suicide attempts recorded at the time of the study. The two latest review studies on mental health and suicidal behaviors included Leigh et al. (2023) on the association between social anxiety and risk of suicidality and O’halloran et al. (2022) on the prevalence of suicidal behaviors in youth with Autism. Leigh et al. (2023) suggested a positive linear relationship between social anxiety and suicidal attempts (correlation coefficient *r:* 0.10) and current suicide risk (pooled correlation coefficient *r:* 0.24). O’Halloran et al. (2022) found an 8.3% prevalence (95% CI: 3.6–18.2%) of suicidal behavior in autistic youth. Miranda-Mendizabal et al. (2019) also found an association between a family history of mental disorders and abuse and the risk of male youth suicide attempts (OR*:* 2.63).

O’halloran et al. (2022), Das Neves Peixoto et al. (2017) and Hauser et al. (2013) explored the association between a range of mental health disorders, including social anxiety, bipolar disorder, Autism, and suicidal ideation. O’halloran et al. (2022)‘s review of 22 studies found a pooled 25.2% (95% CI 18.2–33.8) prevalence of suicidal ideation in autistic youth. Das Neves Peixoto et al. (2017) and Hauser et al. (2013) both explored bipolar disorder as a risk factor for youth suicidal ideation. Das Neves Peixoto et al. (2017) obtained a pooled RR of 2.94 (95% CI: 2.30, 3.78), showing that youth with bipolar disorder were more vulnerable to suicidal ideation (Table 2).

Wang et al. (2022) explored the association between self-harm risk and mental disorders, including depression symptoms, anxiety symptoms, personality disorders, adaption disorders, emotional scale scores, and psychological symptoms. The review of 21 studies found a pooled OR of 1.89 (Table 3).

##### Adolescents with previous suicidality and self-harm

A review of five studies by Miranda-Mendizabal et al. (2019) identified previous suicidal ideation as a significant risk factor for suicidal behavior in both female youth (OR: 4.39) and male youth (OR: 3.97); female youth were almost six times more at risk to attempt suicide if they have had previous suicide attempts (OR: 6.96). A review including 15 datasets and 37,784 participants by Gillies et al. (2018) found that suicide attempts were significantly higher in adolescents who self-harmed (RR: 9.14). From 41 comparisons, Castellví et al. (2017) also reported an over-3-fold risk for those with any previous self-injurious thoughts and behaviors (OR: 3.48, 95% CI: 2.71–4.43), suicide ideation history (OR: 3.26), previous NSSI (OR*:* 2.26), and previous suicide attempts had the largest pooled OR of 5.56. Miranda-Mendizabal et al. (2019) also found an association between a family’s previous suicidal behavior and the risk of female youth suicide attempts (OR: 2.84).

##### Adolescents with concerning behaviors

Two reviews investigated behavior issues such as cannabis use, legal problems, and externalizing symptoms. In one review of three studies, including 13,687 participants, Gobbi et al. (2019) found an association between youth cannabis use and suicide attempts (OR*:* 3.46). The other review by Soto-Sanz et al. (2019) explored the association between externalizing symptoms, including problems related to aggressiveness, inattentiveness, disobedience, and criminal behavior, and youth suicidal behavior from 21 studies. Their finding suggested a 1.59-fold higher risk of suicidal behavior for youth who experience externalizing symptoms and a 2.36-fold risk for youth who experience legal problems (Table 1).

In reviews of behavior issues and suicide ideation, Gobbi et al. (2019) found an association between youth cannabis use and suicidal ideation (OR*:*1.50). Epstein et al. (2020) explored the association between school absenteeism and youth suicidal ideation from 34 studies found a significant pooled OR of 1.20, equivalented to 20% increased risk of suicidal ideation (Table 2).

Epstein et al. (2020) also investigated school absenteeism as a risk factor for self-harm and found a significant pooled OR of 1.37. Wang et al. (2022) explored the association between self-harm risk and behavior issues in adolescents, which included internet addiction, alcohol/substance use, smoking, problematic mobile phone use, having run away from home, suicide attempts, internet/mobile phone abuse, intentional misuse of prescription medications, avoidance, opioid misuse, sedative misuse, and gaming disorder. The review included 21 studies, which resulted in a pooled OR of 2.36. Xiao et al. (2022) explored substance use as a risk factor for self-harm. They found a higher prevalence of self-harm in adolescents with a smoking history (24.7%) versus non-smoking adolescents (10.1%), as well as a higher prevalence of self-harm in adolescents with a history of alcohol consumption (24.4%) versus non-drink adolescents (9.3%) (Table 3).

#### Protective factors

##### School Protective Factors

Two of the included reviews discuss school preventative features as protective factors against youth suicidal behaviors. Marraccini and Brier (2017) investigated school connectedness as a protective factor. The included studies used various measurements ranging from single-item questions to multi-construct measuring instruments. The review, which included ten studies with 57,637 participants, found that school connectedness is associated with reduced reports of suicide attempts in general youth (OR*:* 0.59). Findings were consistent when exploring five studies regarding suicidality in high-risk youth (OR*:* 0.60) and four studies examining sexual minority youth (OR: 0.61). Gijzen et al. (2022) explored the use of school-based interventions (such as Signs of Suicide, Headstrong, Good Behavior Game, and Mastery Learning) with the primary aims of addressing suicidal thoughts and behaviors (STB) or related mental health outcomes, e.g., aggressive, and disruptive behaviors. The review of 5 studies found that prevention of STBs at school was a significant modifier of effect for suicidal behaviors (Corrected effect size Hedges’ *g*: 0.30) (Table 1).

Marraccini and Brier (2017) also explored school connectedness as a protective factor for adolescent suicidal ideation. Their review found a pooled OR of 0.53, indicating that adolescents who experience school connectedness are less likely to experience suicidal ideation. Gijzen et al. (2022) investigated the use of school-based STB interventions. The review of 7 studies with 19,803 participants found that prevention of STBs at school was a significant modifier of effect for suicidal ideation (*g*: 0.15) (Table 2).

##### Optimal Sleep duration

Chiu et al. (2018) investigated sleep duration as a protective factor of adolescent suicidality. They found a pooled OR of 0.52, suggesting a lower risk of suicide attempts from adolescents with longer sleep duration; similarly, a pooled OR of 0.55 and 0.50 for suicidal ideation and ideation with a plan. Of 11 studies with 446,033 participants, their review also identified a significant nonlinear dose-response relationship between the risk of adolescent suicide attempts, indicating that the lowest risk of adolescent suicide attempts was observed with a sleep of 8–9 hours (Table 1). For ideation with a plan, the study found that risk decreased by 11% for every 1-hour increase in adolescent sleep duration (Table 2).

#### Other Exposure Risk Factors

Miranda-Mendizabal et al. (2019) studied several other exposure risk factors for suicidal behavior, including childhood maltreatment, community violence, parental separation, and hopelessness. The review found significant associations between childhood maltreatment and suicidal behavior for female (OR: 2.76) and male adolescents (OR: 3.77) (Table 1). Xiao et al. (2022) found that certain family structure factors can influence adolescent self-harm. They reported that self-harm was more prevalent in adolescents from families with multiple children (27%) than families with one child (25.8%), and self-harm was higher in adolescents from single-parent families (30%) than those from two-parent families. Wang et al. (2022) investigated the association between physical symptoms (which included five factors such as disabilities and sleep problems) and the risk of youth self-harm, reporting a pooled OR of 2.85 (Table 3).

### Results of numerical synthesis

In the results presented in Appendix 2, GSHS across different continents provided an estimated bully victimization prevalence of 30.4% in low- and middle-income countries and an estimated 30.5% in low-, middle- and high-income countries. Based on the same GSHS study, the pooled OR of bully victimization to suicide attempts was 3.06, and the pooled OR representing the global level was 2.97. Accordingly, the bully victimization PAF for suicide attempts was estimated to be 31.4% for low- and middle-income countries and 33.6% for low-, middle- and high-income countries; PAF for suicide ideation was estimated to be 21.8% for low-, middle- and high-income countries. These results indicated that in an ideal environment of no bully victimization, 33.6% of the suicide attempts and 21.8% of the suicide ideation would not have occurred.

The exposure with the second high PAF was sleep disturbance. Based on the sleep disturbance prevalence estimated from the GSHS study and the RR estimated from the meta-analysis, the sleep disturbance PAF for sleep suicide ideation was 12.1% and 10.4% for suicide attempts. These results can also be interpreted as if there is no sleep disturbance in school-age children and adolescents; 12.1% of the suicide ideation and 10.4% of the suicide attempts could have been prevented.

School absenteeism is defined differently in global studies. The most defined problematic school absenteeism includes truancy and school refusal. In educational literature, the acceptable definition of school absenteeism could refer to school-aged youth who (1) have missed at least 25% of total school time for at least two weeks, (2) experience severe difficulty attending classes for at least two weeks with significant interference in a child’s or family’s daily routine, and (3) are absent for at least ten days of school during any 15-week block while school is in session (i.e., a minimum of 15% days absent from school) (Kearney, 2008). Both (1) and (3) include at least 25% missed school days.

In the result of PAFs, school absenteeism PAF was 4.5% for suicide ideation when including both chronic (missing ≥30% school days) and moderate (missing 20–30% school days), according to the New Zealand school attendance service (EducationCounts, 2023). We did not have sufficient data from the literature to provide reliable school absenteeism PAF estimates for suicide attempts.

The PAFs derived using the pooled OR as an approximation for RR yielded a similar range to those using the converted RR, except for a noticeable difference in the PAF of bully victimization for suicidality (Appendix 2).

The meta-analysis included two reviews of 24 studies (Holt et al., 2015; Van Geel et al., 2014) and one school global health survey (Koyanagi et al., 2019) resulted in a pooled OR of bully victimization to suicide attempts being 2.97 (95% C.I. 2.53–3.49, *p* < 0.0001) (Fig. 1). There were significant heterogeneities across the studies, with I^2^ (total heterogeneity / total variability) 86.1% (Fig. 2). These results provided type III suggestive evidence according to the established criteria of epidemiologic credibility (Kohler et al., 2018).

There was no evidence suggesting significant publication bias (p: 0.80, using the Beggar test for asymmetric funnel plot, including year of publication and Countries as the moderators). Sensitivity analysis based on study design, removing the GSGH study had a pooled OR: 2.97(95% C.I. 2.51–3.52).

### Quality assessment and assessment of epidemiologic credibility

The quality assessment of each review used the Joanna Briggs Institute (JBI) “Critical Appraisal Checklist for Systematic Reviews and Research Synthesis (Aromataris et al., 2015). The checklist contains ten questions, and each study was assessed by two reviewers (IZ, T.C.). A consensus was made through discussion and reviewed with a second opinion from a third reviewer (RFR). The median consensus JBI score was 8, with interquartile ranging between 6 and 9. The inter-rater agreement measured by the Cronbach Alpha of internal consistency was 0.68. The sources and resources used to search for and recommend policy and practice presented the most disagreements. All studies were included, and only three studies without meta-analysis of studied outcomes were excluded (Fig. 1).

## Discussion

Adolescent mental health outcomes require urgent attention as suicide continues to be a leading cause of adolescent mortality worldwide (WHO, 2019). Although existing literature has a large number of publications about the risk and protective factors of suicidality and self-harm, a review study describes a whole picture of these factors, and comparing their attributing effects will be helpful for researchers and practitioners to develop guidelines and policies for prevention. This study utilized an umbrella review method with meta-analysis, allowing for a broader view of known risk and protective factors of adolescent suicidality and self-harm, and to obtain an in-depth understanding of those exposure factors relevant to school students. It hopes to provide synthesized information for research and future public health policy in this area.

Data synthesis found that the key factors that play a role in the risk of youth suicidality and self-harm are exposures such as bullying, antidepressants, and sleep disturbance, as well as vulnerabilities including gender, mental health, sexual orientation, previous suicidality, and self-harm. The risk factor findings reflect existing knowledge about youth mental health outcomes, specifically that school-based exposures such as bullying perpetration and victimization can increase the risk of self-harm and suicide behaviors (Islam et al., 2022a; 2022b; Granello et al., 2022) and other exposures such as sleep disturbances and antidepressant use are also associated with such behaviors (Nguyen et al., 2023; Whitely et al., 2020). The study findings also re-iterated these factors’ shared impacts on the risk of suicidality and self-harm of adolescents (Figueiredo et al., 2023; Ougrin, 2014).

While a number of risk factors were found, only two protective factors, school interventions, and optimal sleep duration, were identified. The limited number of protective factors found was unexpected given the emphasis on aspects such as family and peer support as well as cultural identity as protective factors for suicidality and self-harm advocated by the New Zealand government and other health organizations. Despite a lack of prevention strategies highlighted in the included reviews, exploration of New Zealand grey literature emphasizes explicitly the existence and use of established programs which provided more holistic prevention strategies when working with Māori and Pasifika youth. These programs commonly promote components of well-being such as connection, communication, family, cultural identity, and spirituality (Le-Va, 2023).

The results of the data synthesizing, including predominantly school-aged adolescents, gave clearer insight into the role of school with both the risk factors associated and ways to intervene. The risk effects of bullying, sleep, female gender, school absenteeism, and previous self-harm remained observed when limited to the school-age range. The protective factors of school interventions and optimal sleep duration are also identified explicitly for school-age adolescents. These results were further illuminated by the numerical synthesis findings, highlighting the exposure factors of school absenteeism, bully victimization, and sleep disturbances, of which bullying has the highest PAF.

In the literature, the strength-based resilience theory (Zimmerman et al., 2013) provided a conceptual framework to focus on the positive contextual, social, and individual factors that interface with or disrupt the risk of adverse health outcomes in adolescent development. In the current study, the protective factors discovered, such as the sleep optimal hours, were also considered as the promotive factors fitting in the compensatory model because optimal sleep hours, independent from risk factors, were found to reduce the risk of suicide ideation and attempts. School connectedness and school intervention can be considered as either promotive or protective factors depending on their relationships with the risk factors; for example, some school interventions may act as moderators to the association between risk factors and suicidality. Gallagher and Miller’s (2018) ecological framework focused on the protective factors related to the family context, relationships with peers, and the school and community context. The current review identified gaps in a meta-analysis of protective factors related to family attachments, peer relationships, and community factors.

### Limitations and Future Research Implications

Given the focus on school-aged youth, the findings of the study offer insight into what interventions may help target suicidal and self-harm thoughts and behaviors at school. The reviews by Gijzen et al. (2022) and Marraccini and Brier (2017) offer insight into existing school interventions and school connectedness, which have proven effective and may contribute to future interventions for suicidality and self-harm in schools. The findings also highlight the importance of creating a bully-free school environment and a monitoring and responsive environment that encourages attendance. The association between optimal sleep duration and adolescent mental health outcomes may require reflecting on school starting times to allow students to have optimal sleep (Adolescent-Sleep-Working-Group et al., 2014) and intervention to help students develop good sleep patterns. School interventions could also consider promoting health literacy within teaching staff in suicidality and cooperative programs for parents of those in vulnerable populations.

Despite the large number of countries and cultures covered in the current umbrella review, the study failed to capture the prevention-focused holistic view often shared by indigenous cultures (Russell, 2018). This is concerning because indigenous cultures are disproportionately affected by suicidality (Lawson-Te Aho & McClintock, 2020). More generally, several protective factors identified by established organizations, such as the CDCP (2022) and (WHO, 2014), are not covered in the current study and require further exploration. Future research is needed to investigate protective and compensatory factors (Gallagher & Miller, 2018; Zimmerman et al., 2013); qualitative research may give better insight into the role of family, social, cultural, and contextual protective factors. Longitudinal and interventional research investigating protective factors should include resilience-building and interventional programs for bullying victimizers and perpetrators. New Zealand research on bullying prevention programs has been trialed over the last thirty years (Green et al., 2013; Green et al., 2020) and indicates these programs are effective. One such trial is the KiVa program developed in Finland in the late 1990s. It is school-based and for children aged 7 to 15 years. Over three teaching sessions, bullying is considered undesirable, and defending others is desirable. Children are taught to recognize bullying, support victims, and stand up to bullying. In RCTs, KiVa has been indicated to have a solid evidence base (Green et al., 2020). Recent follow-up research asked parents their views of the KiVa program and highlighted the importance of communication between them and the school, as more communication resulted in positive responses (Young et al., 2022).

This review also identified sleep disturbance as a risk factor and optimal sleep duration as a protective factor. Getting enough sleep is considered protective, but poor sleep quality also contributes to mental health disorders; this relationship may be bidirectional (Orchard et al., 2020). Sleep is also interrelated with family issues (Maratia et al., 2023). This interplay highlights that while multifactorial, the evident importance of adolescents, school, family, and community are all relevant in prevention strategies. Researchers should work with health professionals, educationalists, parents, and adolescents to provide high-quality and ongoing prevention programs.

This review highlights the need for further research to elucidate the nature of risk and protective factors, given that there is a pronounced interaction of factors. If good quality sleep is protective, then how can it be enhanced? Recent research into the use of technology by adolescents to enhance sleep indicates that there is a movement to employ all resources, even those that have been previously labeled as risks, such as phone use at night (Daniels et al., 2023). Similarly, in a small study of French adolescents in lockdown during the COVID-19 pandemic, the peer group had less influence than usual, and spending more time with family caused less stress and substance use than they usually experienced (Bourduge et al., 2022). Diverse thinking about technology and developmental norms could be part of the research community’s future contribution to this issue.

Several limitations of the study are worth discussing. Firstly, the umbrella review only included reviews with meta-analysis synthesis. The identified factors are limited to those with numerically synthesized evidence. Secondly, PAF was approximated from OR, with only one factor being used, and without adjustment of multiple variables, its values will be limited to resource and strategy planning comparisons. Secondly, qualitative reviews were not included in this umbrella review, which would further enhance the findings. Thirdly, research studies suggested a bidirectional relationship between psychopathology and sleep. However, these findings were not evident from the current review. Fourthly, the identified school risk factor -school absenteeism is from a meta-analysis of the association and causational effect of this factor on suicidality and self-harm, which requires future research from longitudinal and data integration studies. Fifth, in the review process, the quality assessment was used for description only; the data and numerical synthesis included reviews with moderate quality. Although no significant publication bias was identified from the studies in the meta-analysis, one-third of the reviews included in the narrative data synthesis have not assessed publication bias.

### Implication for policy and schools

Teachers play an essential role in school preventing bullying in school; however, teachers also need to be involved in establishing anti-bullying prevention interventions (Rigby, 2011). Inclusiveness/connectedness and improved bullying interventions are found to be protective factors for adolescent suicide ideation (Marraccini & Brier, 2017). The classroom culture within schools needs to shift from competition to cooperation to create an environment in which students feel connected and have a sense of belonging and safety (Green et al., 2013). Teachers are also required to have mental health literacy skills to be the primary responders.

A solution could be to reset the student-teacher relationship to enable bullying prevention (Green et al. 2013). There is also a suggestion to implement multiple interventions tailored to the situation. For bullying interventions to be effective, the students and the teacher must have mutual regard for each other and their roles in the learning process. A suggestion is to apply multiple individualized strategies to each situation (Burger et al., 2015; Green et al., 2013; Rigby, 2011), for example, the KiVa anti-bullying program (Young et al., 2022).

In New Zealand, the School Start Time Study Advisory Group has recently released a viewpoint article that outlines the biological imperatives of the developmental period of adolescence that make teens stay awake longer in the day and sleep in later in the morning (Barber et al., 2022). They noted the evidence that the “social jet lag” associated with less-than-optimal sleep duration is related to a range of adverse mental health outcomes, including self-harm and suicidality, And suggest later school start times as a public health initiative could be part of a solution to New Zealand’s high rates of youth mental illness, as have been recommended in the US by bodies such as the American Academy of Pediatrics (Barber et al., 2022). Further work is required by the Advisory Group to get their suggestions implemented to improve teens’ sleep duration.

Apart from these abovementioned interventions, programs, and policies, the role of school-based nurses who provided counseling was also found to be associated with strengthened resilience, the capability to manage teasing and bullying, and decreased child anxiety and concentration problems (Best et al., 2018). In 2019, The New Zealand Government announced a youth well-being strategy with measures to reduce bullying (Summary Report - National Engagement on New Zealand’s First). The role of school-based health services was suggested as an intervention in this strategy. This has been implemented since 2020, with the number of school nurses increasing (Hipkins, 2020). Similar measures have been recently reported in New South Wales in an alliance between the Health and Education State Government departments called the Wellbeing and Health In-Reach Nurse Coordinator program https://education.nsw.gov.au/student-wellbeing/whole-school-approach/wellbeing-support#Wellbeing. As a result of these policies, it is to be hoped that the deleterious health effects of bullying on children and youth will be reduced.

## Conclusion

Obtaining knowledge of protective and risk factors is important in adolescents’ suicide and self-harm prevention. There is a lack of research studying their relative attributed impacts and how these factors interact within the resiliency framework. This study used an umbrella review and meta-analysis to synthesize evidence of risk and protective factors of self-harm and suicide attempts in adolescents. To conclude, the broader picture findings of the current review suggest that factors that play a role in youth suicidality are bullying, sleeping disturbance, school absenteeism, and antidepressant exposure. Youth self-harm shared most of these risk factors. Several significant vulnerable young populations were identified with a significantly higher prevalence of suicide attempts and ideation, including lesbian, gay, bisexual, transgender, queer (or questioning) youth and those with mental health disorders, problem behaviors, previous suicidality, self-harm, and gender (female). More specifically for school-age adolescents, the meta-analysis numerical findings suggest that it is vital to create bully-free environments, reduce school-related exposures, and provide protective interventions within schools, offering insight into future public health policy.

## Data Availability

The data supporting this study’s findings are available in the shared repository: https://github.com/suicideprevention/umbrella-review-data-for-synthesis.

## Appendix 1: Quality Appraisals

**Table:**

Studies	Is the review question clearly and explicitly stated?	Were the inclusion criteria appropriate for the review question?	Was the search strategy appropriate?	Were the sources and resources used to search for studies adequate?	Were the criteria for appraising studies appropriate?	Was critical appraisal conducted by two or more reviewers independently?	Were the methods used to combine studies appropriate?	Was the likelihood of publication or any other type of bias assessed?	Were recommendations for policy and/or practice supported by the reported data?	Were the specific directives for new research appropriate?	Total score consensus*
Williams et al. (2021)	1	1	1	1	1	1	1	1	1	0	9
Liu et al. (2019)	1	0	1	1	1	1	0	1	1	1	9
Koyanagi et al. (2019)	1	na	na	na	na	na	1	na	1	1	na
Li et al. (2022)	1	1	1	0	1	1	0	1	1	1	8
Miranda-Mendizábal et al. (2017)	1	1	1	0	1	1	0	1	1	1	8
Miranda-Mendizabal et al. (2019)	1	1	1	0	1	1	0	1	1	1	8
O’halloran et al. (2022)	1	1	1	0	1	1	0	1	1	1	8
Van Meter et al. (2022)	1	0	0	0	0	1	1	1	0	0	6
Marraccini and Brier (2017)	1	1	1	0	0	1	1	1	1	1	9
Dubicka et al. (2010)	1	1	1	1	0	0	1	0	1	1	5
Gili et al. (2019)	1	1	1	1	1	1	0	1	1	0	8
McKinnon et al. (2016)		na	na	na	na	na	na	na	na	na	na
Hauser et al. (2013)	1	1	0	0	0	0	0	0	1	0	3
Leigh et al. (2023)	1	1	1	1	1	1	1	1	1	1	10
Holt et al. (2015)	1	1	1	1	0	0	0	1	1	0	6
Marshal et al. (2011)	1	1	0	0	0	0	1	1	1	1	6
Quarshie et al. (2020)	1	1	1	1	0	0	0	0	0	1	5
Gijzen et al. (2022)	1	1	1	1	1	0	1	1	0	1	8
Gillies et al. (2018)	1	1	0	1	1	1	1	0	1	1	8
Das Neves Peixoto et al. (2017)	1	1	1	1	0	0	0	0	0	1	5
Castellví et al. (2017)	1	1	1	0	0	1	1	1	1	1	8
Gobbi et al. (2019)	1	1	1	1	1	0	1	0	0	1	7
Xiao et al. (2022)	1	1	1	1	1	0	1	1	0	1	8
Chiu et al. (2018)	1	1	1	1	1	1	1	0	1	1	9
Kowalski et al. (2014)	1	1	1	1	0	0	1	0	0	1	6
Heerde and Hemphill (2019)	1	1	1	1	0	0	1	0	1	1	8
Epstein et al. (2020)	1	1	1	0	1	1	1	1	1	1	9
Soto‐Sanz et al. (2019)	1	1	1	0	1	1	1	1	0	1	9
Yang and Feldman (2018)	1	1	1	1	0	0	1	0	0	1	6
Wang et al. (2022)	1	1	1	0	1	0	1	1	1	0	7
Barbui et al. (2009)	1	1	1	0	1	1	1	1	1	1	9
Van Geel et al. (2014)	1	1	1	0	0	0	1	1	1	1	7
Hetrick et al. (2012)	1	1	1	1	1	1	1	1	1	1	10

*The presented total score are results of discussion from three reviewers (I.Z., T.C. and R.F.R.).

## Appendix 2. Estimated PAF of School and School Attendance-Related Risk Factors as Exposure to Suicidality

**Table:**

Exposures Outcomes of Suicidality and NSSI/Self-harm	Prevalence of exposures/pooled prevalence of exposure*	Pooled OR	Prevalence of the mental health outcomes (p0)	RR	PAF (RR est. by OR)	PAF using R.R.	95% C.I. of PAF** (est. by OR)
School absenteeism*							
Suicide ideation	13.85%^1^	1.20	14%^1^	1.17	2.70%	2.26%	0.8–4.9%
	28.80%^2^	1.20	16%^2^	1.16	5.45%	4.48%	1.6–9.6%
	10.90%*^3^	1.20	14%^1^	1.17	2.13%	1.79%	0.6–3.9%
Self-harm (including NSSI)	28.80%^2^	1.37	28%^4^	1.24	9.63%	6.50%	6.0–13.4%
Non-suicidal Self-injuries (NSSI)	28.80%^2^	1.37	18%^4^	1.28	9.63%	7.57%	Na^8^
Bully victimization							
Suicide ideation	30.50%^6^	2.25	14%^1^	1.91	27.60%	21.82%	22.3–30.6%
Suicide attempts	30.40%^5^	3.06	11%^5^	2.51	38.51%	31.42%	
	30.50%^6^	2.97	6%^6^	2.66	37.53%	33.56%	31.6–40.8%
NSSI	30.40%^5^	1.98	18%^4^	1.68	22.95%	17.20%	Na^8^
Sleep disturbances							
Suicide ideation	14.10%^7^	2.35	14%^1^	1.98	15.99%	12.10%	10.3–22.4%
Suicide attempts	14.10%^7^	1.92	6%^6^	1.82	11.48%	10.36%	6.9–16.7%

Based on available prevalence rates of exposure and outcomes from meta-analysis and WHO Global School Health Surveys.

*CI* confidence interval, *OR* odds ratio, *PAF* population attributable factor, *p0* prevalence, *RR* risk ratio.

Footnotes: *Three prevalence rates of school absenteeism were presented to derive the PAF, and the Pooled Odds Ratio of school absenteeism to different outcomes were extracted from the review and meta-analysis of Epstein et al., 2020:

1. The prevalence of 13.85% was derived from New Zealand school attendance registry only, including chronic absence (attending < 70% school days). 14% was the approximated ideation prevalence of the general adolescent population from the Global School-based Student Health Survey (GSHS). OR 1.20 (Epstein et al., 2020).

2. The prevalence of 28.8% was derived from New Zealand school attendance registry, including chronic absence and moderate absence (attending more than 70% up to 80% school days). 14% was the approximated suicide ideation prevalence of the general adolescent population from the GSHS; 16% is the suicide ideation prevalence from GSHS for the Western Pacific region (Biswas et al. 2020a, 2020b). OR 1.37 (Epstein et al., 2020).

3. The pooled prevalence of 10.90% was derived from a global meta-analysis study of chronic school absenteeism (Gubbel et al., 2019).

4. **18**% is an estimated prevalence of NSSI in a meta-analysis for NSSI and DSH (Muehlenkamp et al., 2012). 28% is an estimate of self-harm in the largest Epidemiology study of 11 European countries.

5. The pooled prevalence of 30.4% of bullying victimization and the prevalence of suicide attempt 11% are estimates from GSHS only including low- and middle-income countries (Koyanagi et al. 2019); OR 3.06 is from this same study.

6. The prevalence of 30.5% bullying victimization is an estimate of the Global School Health survey of 83 countries across low-, middle- and high-income countries (Biswas et al. 2020a, 2020b); OR 2.97 of suicide attempts represents pooled meta-analysis results from two review/meta-analysis studies (Holt et al. 2015; van Geel et al. 2014) and the GSHS study including low-, middle- and high-income countries; Prevalence of suicide attempt 6% were derived from the meta-analysis of global prevalence (Lim et al., 2019). Alternatively, OR 2.25 of suicide ideation is the inversed variance weighted geometric mean derived from the review studies (Holt et al. 2015; van Geel et al. 2014).

7. Prevalence rate of sleep disturbance of 14.1% were extracted from the Global school health survey for all high-income countries (Hasan et al., 2023); both pooled O.R.s were extracted from Meta-analysis study of Liu et al. 2019.

8. The confidence interval of PAF for NSSI was not estimated because the log(OR) standard deviation was not available.

** The 95% confidence intervals derivation using the 5% and 95% quartile of the permutated distribution of PAF.
